# Autism-associated biomarkers: test–retest reliability and relationship to quantitative social trait variation in rhesus monkeys

**DOI:** 10.1186/s13229-021-00442-w

**Published:** 2021-07-08

**Authors:** Ozge Oztan, Catherine F. Talbot, Emanuela Argilli, Alyssa C. Maness, Sierra M. Simmons, Noreen Mohsin, Laura A. Del Rosso, Joseph P. Garner, Elliott H. Sherr, John P. Capitanio, Karen J. Parker

**Affiliations:** 1 Department of Psychiatry and Behavioral Sciences, Stanford University, 1201 Welch Rd., MSLS P-104, Stanford, CA 94305 USA; 2 California National Primate Research Center, 1 Shields Ave., Davis, CA 95616 USA; 3 Department of Neurology, University of California, 675 Nelson Rising Lane, San Francisco, CA 94158 USA; 4 Department of Comparative Medicine, Stanford University, 300 Pasteur Dr., Edwards R348, Stanford, CA 94305 USA; 5 Department of Psychology, University of California, 1 Shields Ave., Davis, 95616 USA

**Keywords:** Arginine vasopressin, Autism spectrum disorder, Biomarker, Cerebrospinal fluid, Kinase signaling pathway, Oxytocin, Rhesus macaque, Social trait variation, Social responsiveness scale

## Abstract

**Background:**

Rhesus monkeys (*Macaca mulatta*) exhibit pronounced individual differences in social traits as measured by the macaque Social Responsiveness Scale-Revised. The macaque Social Responsiveness Scale was previously adapted from the Social Responsiveness Scale, an instrument designed to assess social and autistic trait variation in humans. To better understand potential biological underpinnings of this behavioral variation, we evaluated the trait-like consistency of several biological measures previously implicated in autism (e.g., arginine vasopressin, oxytocin, and their receptors, as well as ERK1/2, PTEN, and AKT(1–3) from the RAS-MAPK and PI3K-AKT pathways). We also tested which biological measures predicted macaque Social Responsiveness Scale-Revised scores.

**Methods:**

Cerebrospinal fluid and blood samples were collected from *N* = 76 male monkeys, which, as a sample, showed a continuous distribution on the macaque Social Responsiveness Scale-Revised. In a subset of these subjects (*n* = 43), samples were collected thrice over a 10-month period. The following statistical tests were used: “Case 2A” intra-class correlation coefficients of consistency, principal component analysis, and general linear modeling.

**Results:**

All biological measures (except AKT) showed significant test–retest reliability within individuals across time points. We next performed principal component analysis on data from monkeys with complete biological measurement sets at the first time point (*n* = 57), to explore potential correlations between the reliable biological measures and their relationship to macaque Social Responsiveness Scale-Revised score; a three-component solution was found. Follow-up analyses revealed that cerebrospinal fluid arginine vasopressin concentration, but no other biological measure, robustly predicted individual differences in macaque Social Responsiveness Scale-Revised scores, such that monkeys with the lowest cerebrospinal fluid arginine vasopressin concentration exhibited the greatest social impairment. Finally, we confirmed that this result held in the larger study sample (in which cerebrospinal fluid arginine vasopressin values were available from *n* = 75 of the subjects).

**Conclusions:**

These findings indicate that cerebrospinal fluid arginine vasopressin concentration is a stable trait-like measure and that it is linked to quantitative social trait variation in male rhesus monkeys.

**Supplementary information:**

The online version contains supplementary material available at 10.1186/s13229-021-00442-w.

## Background

All primate species are social for at least some portion of their lifespan [1]. Sociality requires the ability to recognize and remember conspecifics, communicate information effectively, interact in a species-typical manner, and acquire and use knowledge about social relationships that exist between those in one’s social group [2]. Yet, pronounced individual variation in social functioning is evident within non-human primate species [3, 4]. Although this social variation has been well documented, it has not been systematically studied [5].

Introduction of the Social Responsiveness Scale (SRS) facilitated rapid and large-scale assessment of social trait variation in humans [6, 7]. This scale provides a quantitative measure of typical and atypical social functioning in natural social settings [8, 9]. This scale is scored such that higher SRS scores indicate greater social impairment. SRS scores have been shown to be continuously distributed across the general human population [10]. At the extreme of the population distribution, higher SRS scores overlap significantly with a diagnosis of autism spectrum disorder (ASD) [8, 11], a brain disorder characterized by core social cognitive and interaction deficits [12]. This collective evidence has enabled use of the SRS as both a research tool for measuring the presence of autistic traits in members of the general human population [7, 13], and as a clinical screening tool for ASD [8, 9].

The SRS has proven to be a useful measure of social variation in multiple and diverse human societies [14–17]. Given its broad applicability in humans, the SRS has been adapted for use in chimpanzees [18, 19] and rhesus macaques [20] to enable assessment of the presence of similar social traits in these species. Consistent with the human SRS total score [10], chimpanzee SRS and macaque SRS (mSRS) scores are continuously distributed across their respective general populations [18–21]. Moreover, rhesus monkeys observed to be 1.5 standard deviations (SD) above the population mean on naturally occurring non-social behavior are also rated as having a greater burden of autistic-like traits [21].

Very little is known at present about the biological underpinnings of quantitative social trait variation in human and non-human primates. This is because prior biological investigations have typically focused on the development of species-typical social behavior (e.g., parental behavior onset; social bond formation) in rodent species [22]. Nevertheless, several cellular and neuroanatomical signalling pathways implicated in prosocial behavior, ASD, and/or ASD-related syndromes may underlie the regulation of social trait variation.

The first class of biological candidates includes the hypothalamic neuropeptides oxytocin (OXT) and arginine vasopressin (AVP) [23], and the main receptors to which they bind to regulate social behavior, OXTR and AVPR1A [24, 25]. It is well established that these neuropeptides are critical for the expression of prosocial behavior (e.g., affiliative behavior, social bond formation, parental care, social learning, and memory) [26, 27]. Moreover, experimental (pharmacological or genetic) disruption of OXT and AVP brain signaling produces a variety of social behavior impairments in rodents [24, 25, 28, 29]. Deficits in these neuropeptide signaling pathways have also been documented in mouse models of neurogenetic syndromes with high penetrance for ASD (e.g., Fragile X syndrome, Prader-Willi syndrome, CNTNAP2) [30, 31].

The second class of biological candidates includes two main kinase signaling pathways, RAS-MAPK and PI3K-AKT, which include ERK1/2, PTEN and AKT(1–3). ASD is a common comorbidity in rare syndromes (e.g., Costello, Noonan, LEOPARD, and cardio-facio-cutaneous syndromes) caused by mutations in signaling molecules immediately upstream of ERK (MEK, RAF, SPRED1, and RAS) [32, 33]. Similarly, ERK is hyperphosphorylated in Fragile X syndrome patients [34] and implicated in biochemical dysregulation of tuberous sclerosis, another childhood syndrome associated with ASD [35]. In tuberous sclerosis, the TSC1 and TSC2 heterodimeric complex regulates the signaling pathway from the kinase AKT to mTOR and downstream protein synthesis, and this pathway has been implicated in ASD [36]. Similarly, PTEN and PI3K are upstream of these signaling molecules and there is increasing evidence linking both to ASD [37, 38].

To better understand the biological basis of social trait variation in primates, here, we measured mSRS scores in a sample of socially housed rhesus monkeys using a refined and highly reliable version of the mSRS [21], as described below. We next evaluated the trait-like consistency of various biological “readouts” of the AVP, OXT, RAS-MAPK, and PI3K-AKT signaling pathways. Finally, we tested whether any of the reliable biological measures could robustly predict quantitative social trait variation in this species.

## Methods

### Study design

A detailed study timeline is provided in Table 1. Briefly, behavioral observations were conducted over a 15-month period on *N* = 76 rhesus monkeys. Subjects were studied in two cohorts to accommodate project workload, and to ensure that all behavioral observations were conducted during the non-breeding season. (Restricting behavioral observations to the non-breeding season minimizes the potential impact of seasonal changes in macaque social behavior on mSRS score ascertainment.) Cerebrospinal fluid (CSF) and blood samples were collected from all *N* = 76 subjects. In a subset of subjects (*n* = 43), biological samples were collected thrice over a 10-month period. This enabled us to assess the trait-like consistency of our CSF and blood biological measures across multiple time points in a manner not readily achievable in human participants. Collecting biological samples over much of the year also enabled us to examine the stability of these biological measures across the breeding and non-breeding seasons. Finally, experimenters were blinded to monkeys’ mSRS scores during biological sample collection and quantification.

**Table 1:** Timeline of study procedures

Cohort 1	Behavior collection	Sample collection time 1	Sample collection time 2	Sample collection time 3
Start date	06/13/16	09/28/16	01/27/17	06/08/17
End date	08/23/16	10/28/16	07/07/17	07/21/17
*N*	56	56	43	43

Cohort 2	Behavior collection	Sample collection time 1		
Start date	04/20/17	09/28/17		
End date	09/26/17	10/27/17		
*N*	20	20		

Study sample	mSRS-R scores	Biological data time 1	Biological data time 2	Biological data time 3
*N*	76	57 with all biological data; 75 with CSF AVP data	42–43 (depending on the biological measure)	42–43 (depending on the biological measure)

mSRS-R, macaque Social Responsiveness Scale-Revised; CSF, cerebrospinal fluid; AVP, arginine vasopressin

### Subjects and study site

Subjects were *N* = 76 male rhesus monkeys (*Macaca mulatta*), born and reared at the California National Primate Research Center (CNPRC). All subjects lived in mixed age and sex groups of 58 to 145 individuals, with 98.22 ± 24.14 (Mean ± SD) animals per group. Groups comprised 2–18 matrilines (Median: 14 matrilines per group). Each group was housed in a large, outdoor, half-acre (0.19 ha) field corral (30.5 m wide X 61 m deep X 9 m high). Subjects were housed among 16 of the 24 field corrals.

Subjects were tattooed as infants and dye-marked periodically to facilitate easy identification for husbandry- and research-related procedures. Monkeys had ad libitum access to Lixit-dispensed water. Primate laboratory chow was provided twice daily, and fruit and vegetable supplements were provided once weekly. Various toys, swinging perches, and other forms of enrichment in each corral, along with outdoor and social housing, provided a stimulating environment.

Subjects were 3.73 ± 1.17 (Mean ± SD) years old and ranged in age from 1.25 to 6.27 years at the time of study enrollment. Subjects underwent quantitative behavioral data collection as part of a larger research program focused on the biology of macaque social functioning [21]. Eligibility criteria for the parent investigation included: male, 1–7 years of age, socially housed in any of the outdoor field corrals (i.e., not housed indoors in individual cages), medically healthy, and not simultaneously enrolled in another CNPRC project. Preference for study inclusion was given to animals that had been tested as infants in the CNPRC BioBehavioral Assessment Program and which were housed in a field corral with a minimum of five other eligible animals.

Rank was assessed in each corral by behavioral management personnel who recorded aggressive and submissive interactions following food provisioning. Because each corral contained a different number of males, using the raw rank was ineffective as a direct measure that could be compared across all subjects. Thus, rank was calculated as the proportion of males in the group that the focal individual outranked, such that the highest-ranked individual had a value of 1 and the lowest-ranked individual had a value of 0 [39]. Ranks are assessed monthly at CNPRC; thus, we used monkeys’ ranks collected contemporaneously with their mSRS ratings for the purpose of statistical analysis.

### Behavioral data collection

Subjects were observed unobtrusively in their home field corrals. Inter-observer reliabilities of > 85% agreement were established prior to commencing experimental data collection. Each animal was observed for two 10-min focal samples per day (0800–1030 and 1045–1300) over a 2-week period (called a “biweek”). A maximum of eight subjects (range: six to eight), residing in one or two corrals, were observed per biweek per observer. Behavior was recorded at 15-s intervals using instantaneous sampling and time-ruled check-sheets [40]. At the end of a biweek (at least 1 h after the final observation was concluded and no more than 24 h after the last observation), observers rated each subject on the 36-item original mSRS [20], which we modified from a four-point to a seven-point Likert scale (1 = total absence of the trait, 7 = extreme manifestation of the trait) for each item [21]. Prior to final summary, questions written in the infrequent direction were reverse scored such that higher scores always indicated greater impairment. Since only 17 of the original 36 mSRS items exhibit inter-rater and test-rest reliability [21], here we extracted and tabulated ratings for the 17 reliable items, which form the basis of the mSRS-Revised (mSRS-R) [21]. Final summed mSRS-R total scores can range between 17 and 119. Please see Additional file 1 for the mSRS-R instrument.

### Biological sample collection and processing procedures

Subjects in the present study underwent biological sample collection as part of the larger research program. CSF and blood samples were first collected 79.37 ± 33.42 days (range: 21 to 157 days) after mSRS-R score ascertainment in all *N* = 76 monkeys. A complete biological measurement set was available from *n* = 57 of these subjects. A further subset of these monkeys (*n* = 43) underwent sample collection at two additional time points to enable determination of within-individual consistency in the biological measures for the present study. Sampling procedures were the same as those employed in our prior research [41], and identical for each sample collection time point.

Samples were collected between 0800 and 1100 to minimize any potential circadian effects on the biological measurements. Collection of both CSF and blood samples was accomplished within 10–15 min of initial cage entry; only one monkey per day was sampled from the same corral. Briefly, each subject was captured from his home corral, rapidly immobilized with telazol (5–8 mg/kg), and moved to an indoor procedure room. Supplementary ketamine (5–8 mg/kg) was used as needed to facilitate complete immobilization. Immediately following relocation, CSF (2 mL) was drawn from the cisterna magna using standard sterile procedure. CSF samples were immediately aliquoted into 1.5-mL siliconized polypropylene tubes and flash-frozen on dry ice.

Next, whole blood samples (up to 25 mL) were drawn from the femoral vein. Blood was collected at room temperature into EDTA-treated vacutainer tubes for kinase quantification. Samples were spun over a Ficoll-hypaque gradient and mononuclear cells collected from the interface were washed in PBS 2x, pelleted, and solubilized (in 50 mM Tris pH 7.4, 10 mM EGTA, 0.5% NP-40 and protease and phosphatase inhibitor cocktails). Samples were spun to remove insoluable material and then aliquoted. Whole blood was also collected into PAXgene tubes for neuropeptide receptor gene expression. Samples were left at room temperature for 2 h, subsequently transferred to − 20 °C for 24 h, and then transferred to − 80 °C per manufacturer’s guidelines. All biological samples were stored at − 80 °C until quantification.

After sample collection, each subject was prophylactically administered metoclopramide and ketoprofen. Additionally, replacement fluids were given if needed per veterinary guidelines. Subjects were placed in a standard laboratory cage located in a hospital/transition room for recovery overnight, and then returned to their home corrals the next day.

### CSF neuropeptide quantification

CSF OXT and AVP concentrations were quantified using commercially available enzyme immunoassay kits (Enzo Life Sciences, Farmingdale, NY) [41–44]. These kits have been validated for use in rhesus monkeys and are highly specific and exclusively recognize OXT and AVP, respectively, and not related peptides (i.e., the OXT cross-reactivity with AVP is < 0.02% and the minimum assay sensitivity is 15 pg/mL; and the AVP cross-reactivity with OXT is < 0.001% and the minimum assay sensitivity is 2.84 pg/mL). A research scientist performed sample preparation and OXT and AVP quantification following established procedures. CSF samples were directly assayed (without prior extraction) for OXT and AVP. All CSF samples were assayed in duplicate (100 µL per well) with a tunable microplate reader for 96-well format per manufacturer’s instructions. The intra-assay and inter-assay coefficients of variation were 7.75% and 11.78%, respectively, for OXT. The intra-assay and inter-assay coefficients of variation were 7.70% and 14.86%, respectively, for AVP.

### Blood neuropeptide receptor quantification

Measurement of OXTR and AVPR1A mRNA levels was conducted using protocols developed for rhesus monkeys [41]. Total RNA was isolated and purified using a PAXgene blood RNA kit from blood stabilized in PAXgene RNA tubes (Qiagen, CA). The first strand cDNA synthesis reaction was carried out with iScript Reverse Transcription Supermix (Bio-Rad, CA) with a starting RNA quantity of 1 µg in a 20 µl final volume. qPCR was performed to determine gene expression levels of OXTR and AVPR1A using RT^2^ qPCR Primer Assays for Rhesus Macaque OXTR and AVPR1A (Qiagen, CA), and endogenous control (GAPDH, Life Technologies, CA) was used for normalization. qPCR was performed on the QuantStudio 3 Real-Time PCR System (Applied Biosystems, CA) with SYBR Green (Qiagen, CA). cDNA was PCR amplified in triplicate, and Ct values from each sample were obtained using QuantStudio 3 qPCR software. Analyses were conducted using the comparative Ct method (2^−ΔΔCt^) [45].

### Blood kinase signaling quantification

Blood kinase activation levels were determined using previously published protocols [46], which had been optimized for use in rhesus monkeys [41]. Denatured protein (20 µg per lane)  from the previously generated soluble cytoplasmic fraction was electrophoresed through an 8–12% polyacrylamide gradient gel for 2 h at 100 V at room temperature. The gel was electroblotted onto a PVDF membrane for 1 h at 50 V at 4 °C. After blocking, the blot was incubated with primary antibody (Ras, MEK1/2, Phospho-MEK1/2, Erk1/2, Phospho-Erk1/2, Pan AKT and phospho-AKT, PTEN and phospho-PTEN, and GAPDH, Cell Signaling, MA) overnight at 4 °C, washed and followed by fluorescent secondary antibody (Anti-Rabbit IgG, Cell Signaling, MA) incubation for 1 h at room temperature. The blots were visualized using the LI-COR Odyssey Imager (LI-COR Biosciences, NE).

### Statistical analyses

Data were analyzed using JMP Pro 14 (SAS Institute Inc., Cary, NC). We first calculated the test–retest reliability of the neuropeptide and kinase signaling pathway data as a “Case 2A” intra-class correlation coefficient of consistency estimated by a Restricted Maximum Likelihood Mixed Model following [47]. This yields an effect size equivalent to a correlation coefficient [47]. As noted above, three samples were collected from a subset of subjects for this purpose. Subjects were included in this analysis as long as they had at least 2 out of 3 useable time points per biological measure represented in the data; all *n* = 43 monkeys met this inclusion threshold.

We next performed principal component analysis (PCA) to better understand potential collinearities between the reliable biological measures and their relationship to mSRS-R score. This is critical to prevent collinearities from producing false negatives (or less likely, false positives) in our final General Linear Model (GLM) analysis (see below). This approach is an example of best-practice “sensitivity analysis” [48]. Since almost all of the biological measures exhibited high test–retest reliability, we were able to assess *n* = 57 subjects which had a complete set of biological measurements at the first collection time point. Eigenvalues and scree plots suggested a 3-component solution, accounting for 69.1% of the total variance. This was extracted by varimax rotation for principal components. PCA loadings are effect sizes equivalent to the correlation coefficient between the component and the variable [49].

This PCA suggested that CSF AVP concentration and differential neuropeptide receptor gene expression might predict mSRS-R score, whereas the other biological measures appeared unrelated (as assessed by variable loadings). In order to test this hypothesis, we performed a GLM, predicting mSRS-R score, given the reliable biological measures. The analysis controlled for corral, cohort, age, and rank. Blood OXTR and AVPR1A gene expression were highly correlated, and collinear with total and differential neuropeptide receptor gene expression. We therefore included the total neuropeptide receptor gene expression as the sum of the OXTR and AVPR1A gene expression to capture correlated expression of the two genes, and differential neuropeptide receptor gene expression as the difference between OXTR and AVPR1A gene expression to capture relative up or down regulation of these receptors. All of the other reliable biological measures were included in the analysis. This approach conservatively tests each biological measure controlling for all others, and thus identifies variables driving a relationship while excluding variables that are only secondarily correlated. The assumptions of GLM (normality of error, homogeneity of variance, and linearity) were tested graphically. No transformations were required. As noted above, *n* = 57 subjects had complete biological sample sets available at the first collection time point for analysis. However, CSF AVP concentration had been quantified on all *N* = 76 subjects, and an AVP value was available from *n* = 75 of them. This enabled us to run a follow-up analysis in the entire study sample excluding all biological measures except CSF AVP concentration, but including all of the aforementioned blocking factors, in the model.

The advanced methods employed here greatly increase power and reduce sample size (often by 5- or 10-fold) over analyses such as T-Tests or simple regression, while simultaneously also reducing false positives [50–54]. However, no formal a priori power calculations similar to those used for simpler analyses exist for the analyses employed here. As we and others have argued [50, 55, 56], the best approach to a priori power calculations for advanced analyses is Mead’s Resource Equation [57]. Mead’s Resource Equation provides a sample size above which additional subjects will have little impact on power; this size was smaller than the number of subjects we had available for each analysis. We therefore opted to use all subjects for which we had data, noting that these analyses are all suitably powered. Finally, effect sizes for these GLM analyses are reported as partial eta squared (*η*_p_^2^), which is the square of the partial correlation coefficient *r*_p_ for continuous predictors, following best practice for GLMs [50].

## Results

### Autism-associated biological measures demonstrate robust test–retest reliability

All neuropeptide and kinase signaling measures (except phosphorylated AKT/total AKT) showed significant test–retest reliability within individuals across multiple collection time points (Table 2). The test–retest reliability intra-class correlation coefficients ranged from 42.0 to 88.6% for the reliable biological measures. These measurements spanned a 10-month collection period (inclusive of the breeding and non-breeding seasons) and indicate that circannual changes in breeding behavior do not significantly impact the consistency of these measures.

**Table 2:** Within-individual consistency in biological measures

Biological measure	*N*	ICC ± SE	*P*
CSF AVP level (pg/ml)	42	58.1% ± 16.2%	0.0003
CSF OXT level (pg/ml)	43	42.0% ± 13.8%	0.0023
Blood AVPR1A mRNA level (−ΔCT)	43	72.7% ± 18.0%	< 0.0001
Blood OXTR mRNA level (−ΔCT)	43	56.5% ± 16.0%	0.0004
Total neuropeptide receptor gene expression	43	67.3% ± 17.4%	0.0001
Differential neuropeptide receptor gene expression	43	50.6% ± 15.0%	0.0007
Blood p-ERK/ERK ratio	42	42.8% ± 14.2%	0.0027
Blood p-PTEN/PTEN ratio	42	88.6% ± 20.4%	< 0.0001
Blood p-AKT/AKT ratio	43	18.5% ± 11.1%	0.0950

ICC, intra-class correlation coefficient; SE, standard error; CSF, cerebrospinal fluid; AVP, arginine vasopressin; OXT, oxytocin

### Continuous distribution of mSRS-R scores in the general monkey population

**Fig. 1:**
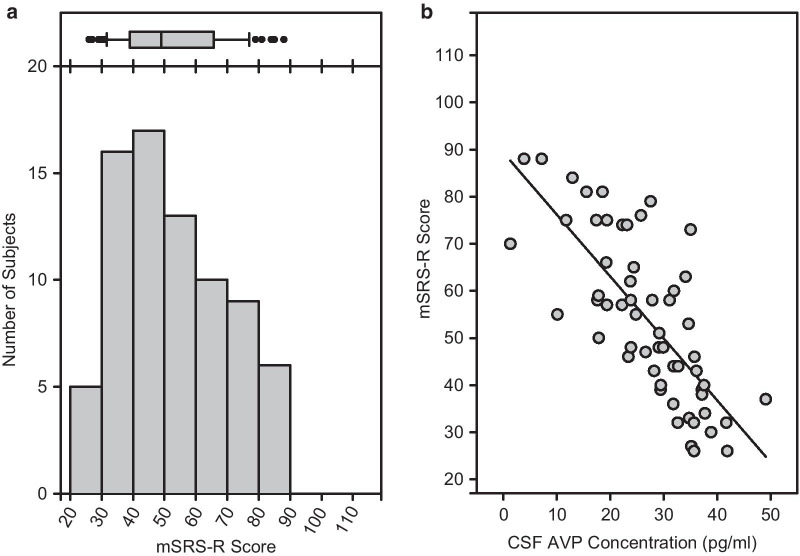
macaque Social Responsiveness Scale-Revised (mSRS-R) scores and cerebrospinal fluid (CSF) arginine vasopressin (AVP) concentration in male rhesus macaques from the general population. **a** mSRS-R scores are plotted as a histogram (*N* = 76), with higher scores indicating greater social impairment. Total mSRS-R scores could range from 17 to 119. A corresponding box-and-whisker plot is shown above the histogram. The box shows the inter-quartile range, the line the median, the whiskers the 10th and 90th percentiles, and the black circles any data out of this range. **b** CSF AVP concentration negatively predicts mSRS-R score (*n* = 57), such that individuals with the lowest CSF AVP concentration demonstrate the greatest social impairment. The observed mSRS-R score is plotted against the partialled CSF AVP concentration. Thus, CSF AVP concentration is corrected (partialled) for the other variables in the analysis and scaled to the range of the original CSF AVP concentration data

Scores on the mSRS-R in this sample ranged from 26 to 88. As observed in past research studies of SRS score distribution in the general human population [10, 58], mSRS-R scores in this sample of rhesus monkeys were likewise continuously distributed across the general population. As also observed in human populations [9, 59], mSRS-R scores were skewed from a normal distribution, indicative of more severe impairment being relatively rare in this monkey species (Fig. 1a). (We note that these mSRS-R data reflect a subset of animals from a larger behavioral study [21], and thus, do not constitute an independent replication sample.)

### A principal component loads mSRS-R score and a subset of autism-associated biological measures

PCA extracted a three-component solution. Only one component loaded mSRS-R score and did so along with two biological measures: CSF AVP concentration and blood differential neuropeptide receptor gene expression. The other biological measures loaded onto two separate components (Table 3).

**Table 3:** Principal component analysis including biological measures and mSRS-R score

Variable	PC1	PC2	PC3
mSRS-R score	0.124757	− 0.068776	− **0.573300**
CSF AVP concentration (pg/ml)	0.128673	0.138965	**0.791405**
CSF OXT concentration (pg/ml)	− 0.051712	**0.895863**	0.105542
Blood AVPR1A mRNA level (− ΔCT)	**0.806013**	− 0.295566	0.310247
Blood OXTR mRNA level (− ΔCT)	**0.949433**	− 0.082827	− 0.268385
Total neuropeptide receptor gene expression	**0.976782**	− 0.176406	− 0.058269
Differential neuropeptide receptor gene expression	− **0.627836**	− 0.121857	**0.599418**
Blood p-ERK/ERK ratio	0.189370	− 0.394305	0.021212
Blood p-PTEN/PTEN ratio	0.028608	− **0.785099**	− 0.129737

Variables with loadings greater than ± 0.4 are considered meaningful (per standard convention) [49] and are noted in bold. Weaker loadings are shown, but greyed out

mSRS-R, macaque Social Responsiveness Scale-Revised; PC, principal component; CSF, cerebrospinal fluid; AVP, arginine vasopressin; OXT, oxytocin

### CSF AVP concentration predicts quantitative social trait variation in the general monkey population

We next used GLM to test the contribution of the biological measures to mSRS-R score. Only CSF AVP concentration significantly predicted mSRS-R score, with lower CSF AVP concentrations predicting higher mSRS-R scores (*F*_1,34_ = 6.629; *P* = 0.0146; *η*_p_^2^ = 16.3%; *r*_p_ = − 40.4%; Fig. 1b). We then confirmed this result by re-running the analysis, but excluding the other biological measures, thereby enabling us to include a greater number of subjects with available CSF AVP values. The result held in the larger study sample (*F*_1,55_ = 4.555; *P* = 0.0373; *η*_p_^2^ = 7.65%; *r*_p_ = − 27.7%).

## Discussion

This study investigated potential biomarkers of quantitative social trait variation in male rhesus monkeys from the general CNPRC population. All biological measures (except AKT) showed significant test–retest reliability within individuals across time points. PCA revealed a potential association between CSF AVP concentration and mSRS-R score; this relationship was subsequently confirmed by GLM (which also indicated that none of the covariates including age and rank, affected mSRS-R scores). Specifically, CSF AVP concentration, but no other biological measure that we tested, robustly predicted individual differences in mSRS-R score, such that monkeys with the lowest CSF AVP concentration exhibited the greatest social impairment. These findings buttress those of a prior study reporting stable within-individual consistency of CSF AVP concentration in a modest sample of *N* = 10 monkeys [41], and add to growing evidence linking individual differences in CSF AVP concentration to individual differences in primate social functioning, including grooming behavior in macaques [41] and Autism Diagnostic Observation Schedule Calibrated Severity Scores in people with ASD [42].

It has been known for nearly three decades that AVP signaling plays a critical role in mammalian prosocial behavior. AVP was first implicated in social bond formation and paternal care in voles [28, 60, 61]. Subsequently, experimental dysregulation of the brain AVP signaling pathway was shown to produce social deficits in mice and voles [24, 28, 29]. Although far less is known about the biology of social functioning in primates, we have shown that naturally low-social adult monkeys, which initiate fewer affiliative interactions, spend less time in physical contact and grooming with conspecifics, and show appreciable social information processing abnormalities (e.g., face recognition deficits and impaired species-typical gaze aversion in response to conspecific aggression) [41, 62–64], exhibit lower CSF AVP concentrations compared to their socially competent peers [41]. Natural silencing of AVP gene activity from birth is also associated with robust social developmental deficits in Brattleboro rats [65, 66], suggesting the intriguing possibility that early impairment in brain AVP signaling may similarly contribute to the pathogenesis of social deficits in young humans.

These findings implicating AVP in mammalian prosocial functioning led us recently to investigate whether CSF AVP concentration is lower in ASD cases vs. controls. This was indeed the case, as in two independent ASD cohorts, CSF AVP concentration accurately differentiated pediatric ASD cases and controls (aged 1.5–19 years). Specifically, across the range of CSF AVP concentrations, the likelihood of ASD increases over 1,000-fold, corresponding to nearly a 500-fold increase in risk with each 10-fold decrease in CSF AVP concentration [41, 42]. We also recently tested the predictive value of AVP by mining over 900 banked CSF samples collected during standard of care from 0 to 3-month-old human newborns. We found that individuals diagnosed with ASD later in childhood have significantly lower neonatal CSF AVP concentrations compared to those who do not later receive an ASD diagnosis [43]. These collective findings suggest that a neurochemical marker of impaired social functioning may be present very early in life, many months or even years, before behavioral symptoms first emerge.

It is well established that ASD is a principally polygenic inherited brain disorder [67], and approximately 100 ASD susceptibility genes have now been identified [68]. Interestingly, AVP itself has not been identified as a high confidence ASD risk gene by SFARI gene, a comprehensive database that catalogues and scores all known human genes for ASD susceptibility [69]. How then might we reconcile findings on AVP’s role in mammalian prosocial functioning [28, 60, 70] with well documented evidence that endophenotypic autistic traits are common, highly heritable, and continuously distributed across the general human population [58, 71]? We suggest that brain AVP signaling may be a downstream pathway critical for the expression of ASD symptoms, and a point of convergence for multiple and diverse ASD susceptibility genes. This would explain our findings linking variation in CSF AVP concentration to variation in grooming behavior in monkeys [41] and clinical symptom severity in ASD patients [42]. This would also explain why intranasal AVP treatment improves social abilities in idiopathic ASD patients, as we recently reported in a double-blind, randomized, placebo-controlled phase 2a pilot trial [72].

## Limitations

This study had several limitations that warrant comment. First, although we used a sophisticated primate model that enabled reverse-translation of a scale that measures social and autistic trait variation in humans, we acknowledge that animal models nevertheless are approximations for examining human neurodevelopmental disease. Second, the relationship between CSF AVP concentration and mSRS-R score in this study was correlational, not causal. Moreover, measurement of AVP in cisternal CSF precluded a more mechanistic understanding of AVP’s role in primate social functioning. Future research using viral vector, optogenetic, gene editing, and/or positron emission tomography radiotracer tools will be required to address this issue in a more tractable manner. Third, unlike the biological measures in this study which were assessed three times, mSRS-R scores were only assessed once per subject, using a relatively new instrument. However, we previously assessed the psychometric properties of the 36-item mSRS, and in the process omitted all items that did not show robust test–retest and inter-rater reliability, resulting in the highly reliable 17-item mSRS-R. We also note that mSRS-R scores are closely related to multiple other measures of social functioning [21]. Fourth, although we assessed biological measures that had been previously implicated in prosocial behavior, ASD, and/or ASD-related syndromes, we did not observe relationships between CSF OXT concentration or blood kinase signaling and social trait variation here. This is in contrast to prior research in humans that has linked neonatal CSF OXT concentration to later social engagement [73], and blood kinase signaling to ASD and its symptom severity [74–76]. However, absence of evidence implicating these biological measures in monkey social functioning is not necessarily evidence of absence. This may be particularly true for the blood kinase signaling measures, as the present study did not capture the theoretical extreme of mSRS-R scores (i.e., the highest mSRS-R score here was an 88 out of a possible 119), and the distribution of scores was statistically skewed toward animals with less social impairment. It therefore remains possible that the relationship between blood kinase signaling and social behavior variation is only evident when studied in a sample of the most socially impaired monkeys. Finally, this study was restricted to male subjects, due to our broader research interest in better understanding the biological underpinnings of ASD, which impacts four times as many males as females [77]. Nevertheless, ASD does affect girls, and it remains to be determined whether the relationship between CSF AVP concentration and social trait variation holds in female subjects.

## Conclusions

In conclusion, this study marked a first step toward better understanding the biological basis of quantitative social trait variation in primates. Findings from this study established the trait-like consistency of nearly all of the biological “readouts” from the AVP, OXT, RAS-MAPK, and PI3K-AKT signaling pathways in rhesus monkeys. Finally, CSF AVP concentration robustly predicted quantitative social trait variation in this species. Research is now required to better understand the role of the AVP signaling pathway in species typical and atypical primate social functioning, and in human primates, AVP’s involvement at the extreme of the social trait distribution, particularly in the pathogenesis of ASD.

## Supplementary information

**Additional file 1.**. macaque Social Responsiveness Scale-Revised Instrument. 

## Data Availability

The datasets used and/or analyzed during the current study are available from the corresponding author on reasonable request.

## Abbreviations

AAALAC International: Association for Assessment and Accreditation of Laboratory Animal Care International
ASD: Autism spectrum disorder
AVP: Arginine vasopressin
CNPRC: California National Primate Research Center
CSF: Cerebrospinal fluid
GLM: General linear model
IACUCs: Institutional Animal Care and Use Committees
ICC: Intra-class correlation coefficient
mSRS: Macaque Social Responsiveness Scale
mSRS-R: Macaque Social Responsiveness Scale-Revised
OXT: Oxytocin
PC: Principal component
PCA: Principal component analysis
SD: Standard deviations
SE: Standard error
SRS: Social Responsiveness Scale
UCSF: University of California, San Francisco
