# Supplementary material for: Autism-associated biomarkers: test–retest reliability and relationship to quantitative social trait variation in rhesus monkeys
Source: Mol Autism. 2021 Jul 8;12:50. doi: 10.1186/s13229-021-00442-w (PMC8268173; doi:10.1186/s13229-021-00442-w)
Supplement: Supplementary file 1 — Additional file 1.. macaque Social Responsiveness Scale-Revised Instrument. [file 13229_2021_442_MOESM1_ESM.docx]

**Supplementary Information**

**The macaque Social Responsiveness Scale-Revised (mSRS-R)**

Instructions: Use your own experience with the animal to form the basis of your rating.

***Please base your ratings upon what is normal for the subjects’ age/sex class.***

Use a seven‑point scale as follows:

1. Displays either total absence or negligible amounts of the trait
2. Displays small amounts of the trait on infrequent occasions
3. Displays somewhat less than average amounts of the trait
4. Displays about average amounts of the trait
5. Displays somewhat greater than average amounts of the trait
6. Displays considerable amounts of the trait on frequent occasions
7. Displays extremely large amount of the trait

1. Seems much more fidgety in social situations than when alone.

total absence 1 2 3 4 5 6 7 extremely large amount

2. Seems self-confident when interacting with others.

total absence 1 2 3 4 5 6 7 extremely large amount

3. Would rather be alone than with others.

total absence 1 2 3 4 5 6 7 extremely large amount

4. Behaves in ways that seem strange or bizarre for others of comparable age/rank/gender categories.

total absence 1 2 3 4 5 6 7 extremely large amount

5. Has good self-confidence.

total absence 1 2 3 4 5 6 7 extremely large amount

6. Does not attempt to interact with other monkeys.

total absence 1 2 3 4 5 6 7 extremely large amount

7. Plays appropriately with peers.

total absence 1 2 3 4 5 6 7 extremely large amount

8. Offers comfort to others when they are sad, e.g., with grooming or other reassuring gestures. Do subjects seek contact with others following a conflict or when they are visibly upset?

total absence 1 2 3 4 5 6 7 extremely large amount

9. Avoids starting social interactions with others.

total absence 1 2 3 4 5 6 7 extremely large amount

10. Is socially awkward. Does not respond appropriately to social cues, e.g., play initiations.

total absence 1 2 3 4 5 6 7 extremely large amount

11. Has overly serious facial expressions, e.g., is not playful (can the subject be described as stoic?).

total absence 1 2 3 4 5 6 7 extremely large amount

12. Is too silly or makes inappropriate noises, e.g., odd vocalizations.

total absence 1 2 3 4 5 6 7 extremely large amount

13. Has repetitive, **odd** behaviors such as hand flapping, rocking/swaying, tumbling or spinning. Displays (motor or self-directed) stereotypical behaviors such as digit sucking, self-clasp, self-hit, self-bite.

total absence 1 2 3 4 5 6 7 extremely large amount

14. Walks in between two monkeys or disrupts them while they are interacting.

total absence 1 2 3 4 5 6 7 extremely large amount

15. Is too tense in social situations, e.g., walks stiffly, stiffens or freezes when others approach. Consider this while accounting for dominance relations. In other words, this should be a generalized behavior, not one that a subject exhibits when in the presence of a higher-ranking individual.

total absence 1 2 3 4 5 6 7 extremely large amount

16. Stares or gazes off into space more so than others of the same age/sex class.

total absence 1 2 3 4 5 6 7 extremely large amount

17. Other monkeys do not like to play with him/her.

total absence 1 2 3 4 5 6 7 extremely large amount
